# Ser500 phosphorylation acts as a conformational switch to prime eEF-2K for activation

**DOI:** 10.1016/j.jbc.2025.111087

**Published:** 2025-12-22

**Authors:** Amanda L. Bohanon, Luke S. Browning, Andrea Piserchio, Kimberly J. Long, Sharon Jacob, Rae M. Sammons, Clint D.J. Tavares, Eun Jeong Cho, Ranajeet Ghose, Kevin N. Dalby

**Affiliations:** 1 Interdisciplinary Life Sciences Graduate Program, The University of Texas, Austin, Texas, USA; 2 Department of Chemistry and Biochemistry, The City College of New York, New York, New York, USA; 3 Division of Chemical Biology and Medicinal Chemistry, The University of Texas, Austin, Texas, USA; 4 Targeted Therapeutic Drug Discovery and Development Program, the University of Texas, Austin, Texas, USA; 5 PhD Program in Biochemistry, The Graduate Center of CUNY, New York, New York, USA; 6 PhD Program in Chemistry, The Graduate Center of CUNY, New York, New York, USA; 7 PhD Program in Physics, The Graduate Center of CUNY, New York, New York, USA

**Keywords:** protein phosphorylation, protein kinase, calmodulin, intrinsic kinase activity, allostery, phosphorus mimetic

## Abstract

Eukaryotic elongation factor-2 kinase (eEF-2K), a member of the α-kinase family of atypical serine/threonine kinases, phosphorylates eEF-2 to slow ribosomal translocation and modulate translational elongation. eEF-2K activation requires Ca^2+^/calmodulin (CaM) and integrates upstream signals through specific sites within an intrinsically disordered regulatory loop (R-loop; ∼321–520) that links the α-kinase core to a C-terminal domain. Unlike canonical CaM-dependent kinases that are activated by displacement of an autoinhibitory segment that occludes the active site, eEF-2K is activated by CaM-driven stabilization of an active state; Ca^2+^/CaM engagement triggers rapid autophosphorylation at T348, which is essential for full activity. Phosphorylation on S500, by eEF-2K or PKA, lowers the CaM requirement (∼20-fold) without increasing maximal catalytic turnover. Here we show that the phosphomimetic S500D markedly enhances CaM binding in the T348-phosphorylated enzyme. S500D also elevates CaM-independent (“intrinsic”) activity even in the absence of phosphorylation at T348, although maximal activity requires modification at both sites. Hydrogen–deuterium exchange mass spectrometry reveals CaM-dependent conformational changes near S500, consistent with relief of inhibitory constraints. Deletion of residues near S500 mimics S500D, increasing intrinsic activity and CaM binding *in vitro* and enhancing eEF-2 phosphorylation in cells, supporting an inhibitory role for this segment. Prior studies have linked S500 phosphorylation to eEF-2K degradation, suggesting a dual regulatory role. We demonstrate that phosphorylation at T348 and S500 synergize to stabilize an active-like conformation and increase CaM responsiveness, effectively lowering the Ca^2+^/CaM threshold for eEF-2K activation and enabling the integration of Ca^2+^, cAMP/PKA, and metabolic cues.

Protein synthesis is one of the most energy-intensive processes in the cell, and its regulation is crucial for maintaining energy homeostasis and enabling adaptive responses to environmental changes (1). Cells fine-tune global translation rates and selectively control the translation of specific mRNAs to reshape the proteome in response to physiological needs (2, 3). A key regulatory node in this process is translation elongation, which, in most eukaryotes, is primarily modulated by eukaryotic elongation factor 2 kinase (eEF-2K) (4, 5). By phosphorylating elongation factor 2 (eEF-2), eEF-2K inhibits ribosomal translocation, thereby slowing elongation (6). This mechanism is thought to conserve energy, enhance translational fidelity, and prioritize the translation of stress-responsive mRNAs (7, 8). In neurons, eEF-2K plays a crucial role in synaptic plasticity, and its dysregulation has been linked to several neurological disorders (9, 10, 11, 12).

eEF-2K is an atypical serine/threonine kinase of the α-kinase family, regulated by calmodulin (CaM) in response to intracellular Ca^2+^ signals, through a mechanism that is distinct from that of canonical CaM-dependent kinases (4, 13). In contrast to kinases such as CaMKI, CaMKII, or MLCK, where Ca^2+^/CaM binding primarily relieves autoinhibition by displacing a regulatory segment to expose or otherwise remodel the active site (14, 15, 16), eEF-2K is activated by CaM through stabilization of an active α-kinase conformation. Ca^2+^/CaM binds to a defined N-terminal CaM-targeting motif (CTM) and triggers rapid autophosphorylation at T348, a modification that is essential for full activation (17, 18). Phosphorylation at a second regulatory site, S500, further stabilizes the active conformation and lowers the CaM concentration required for activity (17, 18, 19). These features enable eEF-2K to respond not only to Ca^2+^ signals but also to metabolic cues and post-translational inputs that favor the enzyme's active state.

Beyond Ca^2+^/CaM binding, eEF-2K integrates regulatory signals from various pathways, including mTOR and PKA, as well as cellular stress, nutrient status, and pH, through specific phosphorylation events (20, 21, 22, 23, 24, 25, 26). These phosphorylation sites are concentrated within a long, intrinsically disordered segment known as the regulatory loop (R-loop), which roughly spans residues 321 to 520 and links the C-terminal end of the α-kinase domain to a conserved helical region that forms part of the functional scaffold designated the C-terminal domain (CTD). The R-loop phosphorylation sites include S500, which is phosphorylated by both eEF-2K itself and PKA (18, 24, 25, 27). While S500 has been shown to promote activation, the broader roles of R-loop phosphorylation in eEF-2K regulation remain incompletely understood.

Here, we investigated how phosphorylation at S500 influences the activation and regulation of eEF-2K, building on our previous discovery that S500 phosphorylation accelerates T348 autophosphorylation and enhances the sensitivity of eEF-2K to CaM, lowering its *K*_CaM_ (the concentration of CaM that elicits half-maximal activation) by approximately 20-fold without altering the catalytic turnover (*k*_cat_) of the CaM-bound complex (19). In this work, we examined how these effects manifest in both unphosphorylated and T348-phosphorylated states of eEF-2K. Using biochemical, biophysical, and cellular approaches, we demonstrate that the phosphomimetic S500D mutation (18, 19, 27) in combination with T348 phosphorylation markedly enhances CaM binding and confers CaM-independent activity, likely by relieving inhibitory constraints. Together, our findings support a model in which initial CaM binding triggers activation, while phosphorylation at T348 and S500 cooperatively stabilizes the active state and enhances the interaction with CaM. These findings extend our previous work and suggest that S500 phosphorylation, mediated by PKA or autophosphorylation, acts as a key signal integrator, enabling basal eEF-2K activity under low-Ca^2+^ conditions and priming the kinase for rapid reactivation upon elevated Ca^2+^ levels.

## Results

### S500D and T348 phosphorylation combine to enhance the calmodulin affinity of eEF-2K

To investigate how phosphorylation at S500 influences eEF-2K function, we utilized an S500D mutant form of the enzyme (S500D-eEF-2K), previously shown to mimic key functional effects of S500 phosphorylation *in vitro* and in cells, including enhanced T348 autophosphorylation, reduced *K*_CaM_, and elevated activity under basal (low Ca^2+^) conditions (19, 27). To test the impact of this modification on CaM binding, we employed native gel electrophoresis using IAEDANS-labeled CaM (CaM_IAE_), as previously described (28). We selected this approach because it provides a sensitive, solution-phase, and state-resolved readout of CaM–eEF-2K complex formation, avoiding artifacts that can arise in SPR or intensity-based methods when analyzing tight or multivalent interactions.

**Figure 1:**
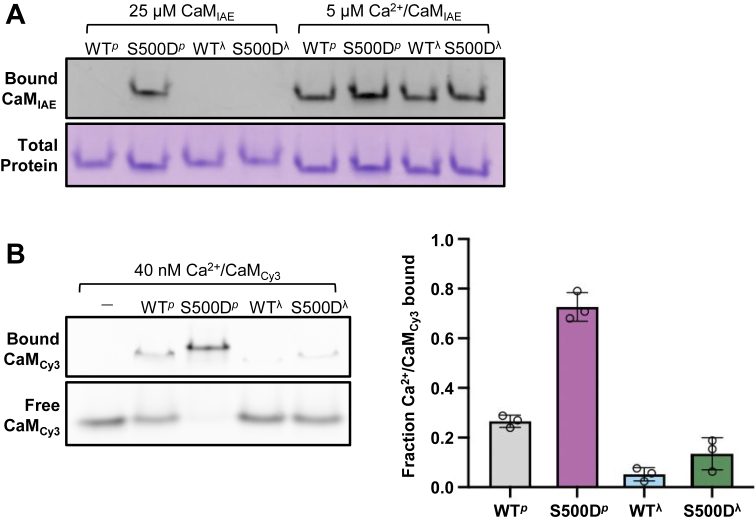
**Dual modification of the eEF-2K regulatory loop at T348 and S500 enhances CaM binding.***A*, the ability of recombinant full-length wild-type eEF-2K (WT) and the phosphomimetic S500D mutant to bind calmodulin (CaM) was assessed using CaM labeled with the fluorophore IAEDANS (CaM_IAE_) on non-denaturing gels. WT and S500D were expressed either in the presence of λ-phosphatase (denoted ^“λ”^; dephosphorylated at T348) or in its absence (denoted ^“*p*”^; phosphorylated at T348), generating four regulatory-loop states (WT^λ^, S500D^λ^, WT^*p*^, and S500D^*p*^). The purified proteins were incubated with the indicated concentrations of CaM_IAE_ under either apo conditions (no added Ca^2+^) or Ca^2+^-bound conditions (+Ca^2+^; 150 μM free Ca^2+^). Samples were resolved on native gels to separate free CaM_IAE_ from eEF-2K•CaM_IAE_ complexes. Bound CaM_IAE_ was visualized by fluorescence, and total protein by Coomassie staining. Under Ca^2+^-bound conditions, CaM_IAE_ binding was highest for the dual-modified S500D^p^ species, consistent with cooperative enhancement of CaM association by T348 phosphorylation and S500D. *B*, *Left*, Ca^2+^/CaM binding was quantified using a Cy3-labeled CaM variant (CaM_Cy3_; CaM_A2C_ labeled at Cys2). The indicated eEF-2K constructs (60 nM) were incubated with 40 nM CaM_Cy3_ at ∼50 μM free Ca^2+^, and complexes were resolved on native gels. Bound and free CaM_Cy3_ were visualized in the Cy3 channel. *Right*, the fraction of CaM_Cy3_ bound (mean ± SD, n = 3) was obtained by densitometry as the fluorescence in the bound band divided by total lane fluorescence. Data were analyzed by two-way ANOVA with factors T348 phosphorylation (λ vs *p*) and S500 substitution (WT vs S500D). T348 phosphorylation had a significant effect, increasing Ca^2+^/CaM binding (*p* < 0.0001), whereas S500D alone did not. A significant interaction between T348 phosphorylation and S500D indicated that S500D enhanced Ca^2+^/CaM binding specifically when T348 was phosphorylated, with little or no effect in the dephosphorylated (λ) state. Post hoc pairwise comparisons were performed using Tukey’s HSD test (α = 0.05). These results support a model in which S500 phosphorylation (mimicked by S500D) cooperates with T348 phosphorylation within the regulatory loop to increase the affinity of eEF-2K for Ca^2+^/CaM, thereby priming the kinase for activation.

Recombinant eEF-2K purified from *Escherichia coli* was fully phosphorylated at T348 (eEF-2K^*p*^), whereas co-expression with λ-phosphatase yielded the unphosphorylated form (eEF-2K^λ^). Native gel analysis showed robust binding of apo-CaM_IAE_ (Ca^2+^-free) to S500D-eEF-2K^*p*^ (S500D^*p*^, Figs. 1*A*, S1*A*). We focused on apo-CaM in this assay to investigate how S500D and T348 phosphorylation affect CaM binding under low-Ca^2+^ conditions, which reflect cellular states where Ca^2+^ levels are reduced but eEF-2K remains active. Although apo-CaM binds more weakly than Ca^2+^/CaM, we have shown that it can nonetheless fully activate eEF-2K at saturating concentrations (19). Given that cytosolic CaM is abundant (∼2–25 μM), and apo-CaM predominates at resting Ca^2+^ levels (29), this state is likely to be physiologically relevant, especially when both S500 and T348 are phosphorylated. In contrast, binding was undetectable for eEF-2K^λ^ (WT^λ^), S500D-eEF-2K^λ^ (S500D^λ^), and eEF-2K^*p*^ (WT^*p*^). Previously, we showed that the predominant role of Ca^2+^ in eEF-2K activation is to promote the binding of CaM to eEF-2K, as it enhances CaM-mediated activation of eEF-2K by ∼1000-fold but has minimal effect on kinase activity once bound (19). To demonstrate that all the above forms of eEF-2K are capable of binding CaM in its high-affinity, Ca^2+^-bound state, we measured the binding of 5 μM CaM_IAE_ in the presence of an excess (150 μM) of Ca^2+^. As expected, the mutation of S500 or phosphorylation of T348 did not affect the binding of eEF-2K to Ca^2+^/CaM under these saturating conditions (Figs. 1*A*, S1*A*).

To assess the effect of these two modifications (phosphorylation on T348 and S500, the latter mimicked by the S500D mutation) of eEF-2K on its binding to Ca^2+^/CaM at physiologically relevant concentrations, we utilized a more sensitive fluorophore, Cy3. To tag CaM with Cy3, we used Cy3-maleimide, which conjugates the dye to thiol groups of proteins. Since CaM does not contain any cysteine residues, we introduced a substitution mutation of alanine two to cysteine in CaM (CaM_A2C_), which did not affect the ability of CaM to stimulate kinase activity. Incubation of CaM_A2C_ with Cy3-maleimide resulted in Cy3-tagged CaM (CaM_Cy3_). Like the binding of apo-CaM_IAE_ (Fig. 1*A*), at 40 nM CaM_Cy3_ and saturating Ca^2+^ (50 μM), the dually modified species, S500D-eEF-2K^*p*^, demonstrated the most pronounced effect on Ca^2+^/CaM binding compared to the unmodified enzyme, eEF-2K^λ^ (Figs. 1*B*, S1*B*). T348 phosphorylation, but not the introduction of S500D into the unphosphorylated enzyme, independently increased CaM association. However, a two-way ANOVA revealed that the effect was greater than additive, indicating a positive interaction (*p* value < 0.001) between the two modifications. This finding aligns with our catalytic measurements, in which S500D lowers *K*_CaM_ only after T348 phosphorylation, without increasing *k*_cat_ (19), consistent with the stabilization of the active state.

### S500D and T348 phosphorylation together enhance the intrinsic activity of eEF-2K

**Figure 2:**
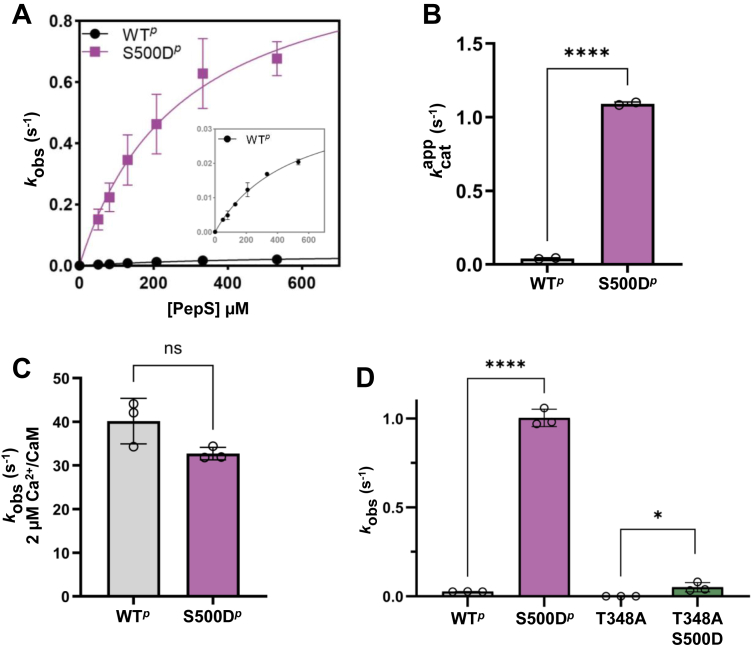
**S500D enhances the catalytic activity of eEF-2K.***A*, the CaM-independent, “intrinsic” activities of eEF-2K^*p*^ (WT^*p*^) and S500D-eEF-2K^*p*^ (S500D^*p*^) were measured in the absence of CaM using a peptide substrate (PepS) in duplicate (n = 2). The observed rate constants, *k*_obs_, were plotted as a function of PepS concentration and fitted to the Michaelis-Menten equation. The inset shows a magnification of the y-axis for the eEF-2K^*p*^ curve. The estimated $K_{m}^{\text{app}}$ and $k_{\text{cat}}^{\text{app}}$ values were 499 ± 86 μM and 0.04 ± 0.004 s^−1^, respectively, for eEF-2K^*p*^ and 275 ± 73 μM and 1.1 ± 0.1 s^-1^ for S500D-eEF-2K^*p*^. *B*, The $k_{\text{cat}}^{\text{app}}$ values for duplicate (n = 2) experiments from (*A*) are displayed as bar graphs. The “∗∗∗∗” indicates *p* < 0.0001 in an unpaired *t* test. *C*, The Ca^2+^/CaM-dependent activities of eEF-2K^*p*^ and S500D-eEF-2K^*p*^ were measured using 450 μM PepS in triplicate (n = 3) to obtain *k*_obs_ values of 40 ± 5 s^−1^ and 33 ± 2 s^−1^, respectively. The “ns” indicates no significant difference (*p* value of 0.0751) in an unpaired *t* test. *D*, the intrinsic activities of eEF-2K^*p*^, S500D-eEF-2K^*p*^, T348A-eEF-2K (T348A), and S500D/T348A-eEF-2K (T348A/S500D) were assessed at a single peptide substrate concentration (450 μM PepS). The bar graphs display the average of the observed rate constants, *k*_obs_, measured in triplicate (n = 3), which were 0.03 ± 0.005 s^−1^, 1.0 ± 0.06 s^−1^, 0.0007 ± 0.0002 s^−1^, and 0.05 ± 0.03 s^−1^, for eEF-2K^*p*^, S500D-eEF-2K^*p*^, T348A-eEF-2K, and S500D/T348A-eEF-2K, respectively. The “∗∗∗∗” indicates *p* < 0.0001 while “∗” represents a *p* value of < 0.05 in an unpaired *t* test.

We previously reported that the S500D mutation enhances the rate of autophosphorylation at T348 by approximately sixfold, suggesting that this modification affects the enzyme’s intrinsic properties beyond simply increasing its CaM affinity (19). To examine whether S500 phosphorylation alters eEF-2K’s inherent catalytic activity, we compared the CaM-independent (intrinsic) activities of eEF-2K^*p*^ and S500D-eEF-2K^*p*^ using a peptide substrate PepS (Fig. 2, *A* and *B*; Table 1). Although the S500D mutant showed no significant change in substrate affinity ($K_{m}^{\text{app}}$), it exhibited a ∼25-fold higher intrinsic activity ($k_{\text{cat}}^{\text{app}}$ = 1.1 s^-1^) than eEF-2K^*p*^ ($k_{\text{cat}}^{\text{app}}$ = 0.04 s^-1^). Consistent with previous findings, the S500D mutation did not affect eEF-2K’s activity (*k*_obs_) in the presence of saturating Ca^2+^/CaM (Fig. 2*C*; Table 1).

**Table 1:** Kinetic parameters for recombinant wild-type (WT) and mutant eEF-2K proteins

		**k**_obs_^b^	$k_{\text{cat}}^{\text{app}}$^c^	$K_{m}^{\text{app}}$^c^
eEF-2K^a^	Calmodulin^a^	s^−1^	s^−1^	μM
WT^*p*^	−	0.03 ± 0.005^d^	0.04 ± 0.004	499 ± 86
	+	40 ± 5	ND^e^	ND
S500D^*p*^	−	1.0 ± 0.06^d^	1.1 ± 0.1	275 ± 73
	+	33 ± 2	ND	ND
T348A	−	0.0007 ± 0.0002	ND	ND
S500D/T348A	−	0.05 ± 0.03	ND	ND
Δ497–502^*p*^	−	0.8 ± 0.1	ND	ND

a Recombinant eEF-2K proteins contain autophosphorylation at T348, denoted as “^*p*^”.

a Experiments contained 0 or 2 μM CaM. All contained ∼34 μM free Ca^2+^.

b Rate constants *(k*_obs_) determined in triplicate with 450 μM peptide substrate and 1 mM γ-^32^P-ATP.

c Apparent catalytic constants ($k_{\text{cat}}^{\text{app}})$ and apparent Michaelis-Menten constant $\left(K_{m}^{\text{app}}\right)$ values were determined from fitting peptide dependence curves to Equation 1.

d Mean ± SD; n = 2 independent experiments (each in technical triplicate).

e ND: not determined.

To determine whether S500D can enhance intrinsic activity in the absence of T348 phosphorylation, we analyzed the T348 A mutant, which was previously shown to markedly reduce CaM-stimulated activity and increase the *K*_CaM_ by ∼7-fold (17). In the CaM-free state, the T348A/S500D double mutant displayed a ∼20-fold lower intrinsic activity than S500D-eEF-2K^*p*^. However, its activity was still ∼71-fold higher than that of T348A alone (Fig. 2*D*; Table 1).

These results demonstrate that S500D enhances intrinsic catalytic activity independently of T348 phosphorylation. However, maximal CaM-independent activity is achieved only when both T348 and S500 are phosphorylated. Remarkably, the CaM-independent activity of the dual-modified enzyme (S500D-eEF-2K^*p*^) is only ∼33-fold lower than its fully Ca^2+^/CaM-stimulated activity (Table 1) and ∼1400-fold higher than that of T348A, underscoring the substantial capacity of these modifications to promote CaM-independent activity and stabilize the active kinase conformation.

### CaM binding and S500D independently disrupt an inhibitory region surrounding S500

**Figure 3:**
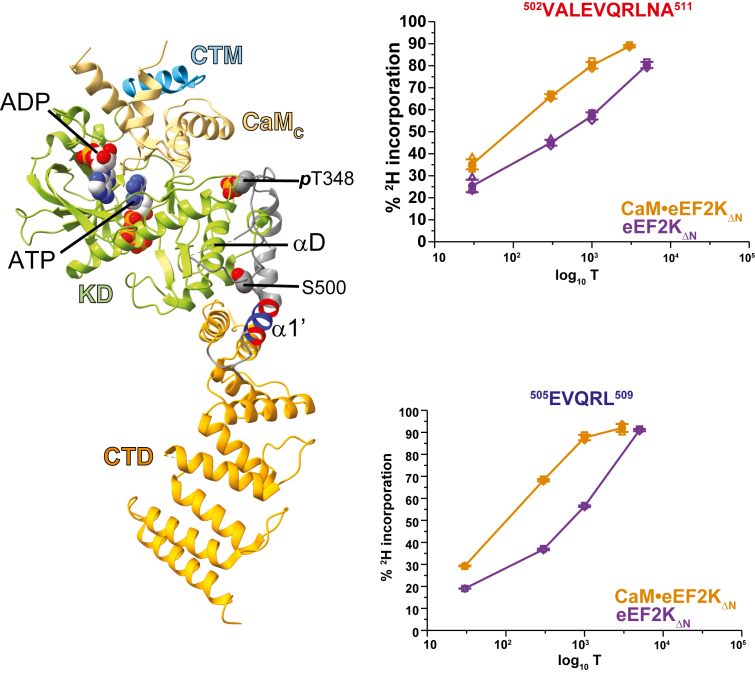
**Hydrogen deuterium exchange mass spectrometry measurements suggest increased R-loop exposure upon CaM binding.** Hydrogen-deuterium exchange mass spectrometry (HXMS) experiments were performed on eEF-2K_ΔN_ alone or in a complex with CaM (CaM•eEF-2K_ΔN_). T348 was unphosphorylated in all cases. The only region within eEF-2K_ΔN_ that showed statistically significant differences between its CaM-free and CaM-bound states involved the V502-D513 segment proximal to S500. Two overlapping peptides corresponding to this region are shown. These peptides illustrate reduced ^2^H incorporation in the CaM-free compared to the CaM-bound state. The left panel shows the location of the peptides on the crystal structure of CaM•eEF-2K_TR_ complex (PDB: 8FNY). Key structural modules—the CaM-targeting motif (CTM, *blue*), the α−kinase domain (KD, *green*), and the C-terminal domain (CTD, *orange*). An ATP molecule bound at the catalytic site, and an ADP bound to the interface between the C-lobe of CaM (CaM_C_, *yellow*) module and the N-lobe of the KD, are shown as *spheres*. The phosphorylated T348 (*p*T348) and the unphosphorylated S500 are also shown. These two residues lie at the two extremes of the αD helix (indicated) with the *p*T348 engaging the N-terminus and S500 engaging its C-terminus. The two peptides, V502-A511 (red) and E505-L509 (*blue*), with the former encompassing the latter, derive from a region on the R-loop that forms a helix (α1′ in the definition of Piserchio *et al.* (30)) in most structures of the CaM•eEF-2K_TR_ complex solved thus far.

Our previous hydrogen/deuterium exchange mass spectrometry (HXMS) analysis of a truncated eEF-2K construct (eEF-2K_TR_; Fig. S2) in complex with Ca^2+^/CaM revealed reduced deuterium uptake near the CaM-binding site in the N-lobe of the kinase domain and around the phosphate-binding pocket (PBP) engaged by phosphorylated T348 (30). Notably, Ca^2+^/CaM binding alone, even in the absence of T348 phosphorylation, conferred protection in and around the PBP (31), indicating that CaM binding induces broader conformational changes. To further explore how CaM affects eEF-2K dynamics, we performed HXMS on an eEF-2K construct containing an intact R-loop but lacking 70 N-terminal residues (eEF-2K_ΔN_, unphosphorylated at T348; Fig. S2) in the presence and absence of Ca^2+^/CaM. Among the regions with sufficient peptide coverage, the only statistically significant change in deuterium uptake occurred in the segment encompassing residues V502–D513, immediately adjacent to S500. In this segment, apo-eEF-2K_ΔN_ exhibited lower deuterium incorporation than the CaM-bound form (Fig. 3), indicating increased solvent accessibility upon CaM binding and suggesting a conformational rearrangement around S500.

**Figure 4:**
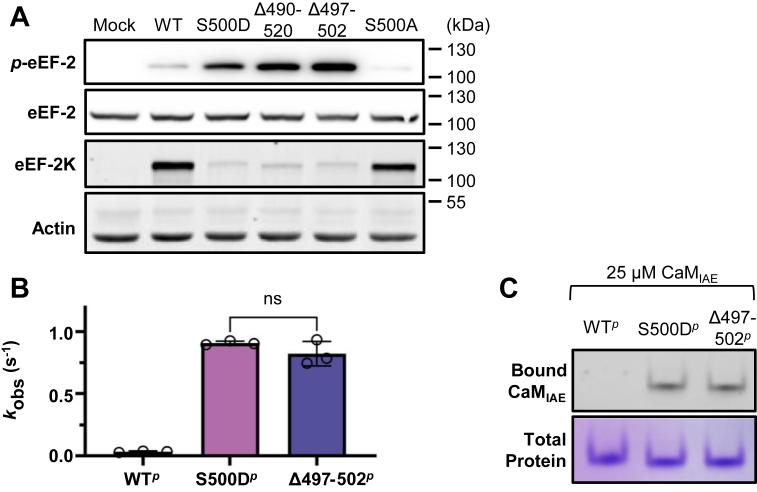
**Activation of eEF-2K is associated with disruption of a region around S500.***A*, representative Western blot of eEF-2K expression and eEF-2 phosphorylation in MCF10A *eef2k*^−/−^ cells transfected with the indicated eEF-2K mutants, performed in two independent biological replicates. *B*, the intrinsic (CaM-independent) activities of eEF-2K^*p*^, S500D-eEF-2K^*p*^, and eEF-2K_Δ497-502_^*p*^ (Δ497–502^*p*^) were measured (in triplicate, n = 3) using 450 μM PepS. *k*_obs_ values of eEF-2K^*p*^, S500D-eEF-2K^*p*^, and eEF-2K_Δ497-502_^*p*^ are displayed as bar graphs. The values in the three cases were 0.03 ± 0.005 s^−1^, 1.0 ± 0.06 s^−1^, and 0.8 ± 0.1 s^−1^, respectively. The “ns” indicates no significant difference (*p* value of 0.2113) in an unpaired *t* test. *C*, the association of recombinant eEF-2K^*p*^ and corresponding mutants with the fluorescent Ca^2+^-free CaM_IAE_*via* native gel.

These observations led us to hypothesize that the region surrounding S500 acts as an inhibitory element displaced upon Ca^2+^/CaM binding or S500 phosphorylation. To test this further, we analyzed S500D-eEF-2K and two deletion mutants in mammalian cells (Fig. 4*A*). Expression of S500D-eEF-2K in eEF-2K-deficient MCF10A cells (MCF10A *eef2k*^*−/−*^) increased eEF-2 phosphorylation, despite substantially reduced eEF-2K protein levels, consistent with previous reports of reduced stability of this mutant (19, 32). Similarly, the mutant in which residues N490–K520 were replaced with a 6-glycine linker (eEF-2K_Δ490–520_; Fig. S2) showed elevated eEF-2 phosphorylation despite lower protein levels. A more conservative alteration, in which residues P497–V502 were replaced with six glycine residues (eEF-2K_Δ497–502_; Fig. S2), also mimicked the effects of S500D (Fig. 4*A*).

To characterize eEF-2K_Δ497–502_ biochemically, we expressed and purified this mutant to assess its CaM-independent intrinsic activity against PepS *in vitro*. The T348-phosphorylated form (eEF-2K_Δ497–502_^*p*^) exhibited intrinsic activity (*k*_obs_ = 0.8 s^-1^) comparable to S500D-eEF-2K^*p*^ (*k*_obs_ = 1.0 s^-1^) and substantially higher than eEF-2K^*p*^ (0.03 s^-1^) (Fig. 4*B*; Table 1). Additionally, eEF-2K_Δ497–502_^*p*^ bound apo-CaM similarly to S500D-eEF-2K^*p*^ (Fig. 4*C*), indicating that this construct recapitulates key biochemical features of the dually modified enzyme.

Collectively, these results support a model in which disruption of the region surrounding S500 at the C-terminal end of the R-loop, through phosphorylation, mutation, or deletion, relieves an inhibitory constraint, enabling partial activation of eEF-2K, and enhancing its affinity for CaM. Precisely how this rearrangement occurs at a structural level remains to be elucidated. The HXMS-detected increase in solvent accessibility at V502–D513 upon CaM binding suggests that this region forms part of an inhibitory element whose displacement is necessary for stable CaM association. S500 phosphorylation or deletion of nearby residues likely increases local flexibility or alters electrostatic interactions within the R-loop, reducing steric hindrance at the CaM-binding interface. This structural change would lower the energetic barrier for CaM binding, explaining the reduced *K*_CaM_ observed for S500D, particularly in the T348-phosphorylated enzyme.

## Discussion

eEF-2K integrates diverse cellular cues, including Ca^2+^ signals and phosphorylation events, to regulate translation elongation. Our study reveals that phosphorylation at S500, a site targeted by both PKA (24, 25, 27) and autophosphorylation (18), fine-tunes eEF-2K’s responsiveness to Ca^2+^/CaM by stabilizing a partially active kinase conformation. This modification enhances the enzyme’s intrinsic (CaM-independent) activity and, particularly when T348 is phosphorylated, increases its sensitivity to CaM, positioning S500 as a regulatory switch that dynamically adjusts kinase activity in response to cellular conditions.

Our findings suggest that S500 phosphorylation lowers the threshold for activation by Ca^2+^/CaM, enabling eEF-2K to respond to otherwise sub-threshold signals. Mechanistically, S500 phosphorylation does not increase the maximal catalytic activity of eEF-2K once fully saturated with Ca^2+^/CaM (similar *k*_obs_ values, see Table 1) (19). Instead, it promotes the formation and stability of the active CaM•eEF-2K complex, particularly when combined with T348 autophosphorylation. The doubly modified enzyme (S500D/T348-phosphorylated) exhibits a ∼1400-fold increase in CaM-independent intrinsic activity and a ∼100-fold increase in CaM sensitivity (lower *K*_CaM_) compared to the T348A mutant (17, 19). Together, these observations indicate that S500 phosphorylation and T348 autophosphorylation stabilize an active conformation of eEF-2K and enhance its responsiveness to cellular signals.

HXMS studies reveal that Ca^2+^/CaM binding increases solvent accessibility in the region adjacent to S500, suggesting a conformational rearrangement. Deletion of S500 and nearby residues mimics the effects of an S500D mutation, resulting in robust CaM-independent activity and elevated eEF-2 phosphorylation, despite reduced eEF-2K protein levels. In the various structures of the CaM•eEF-2K_TR_ complex solved thus far, a helical segment (αD in the definition of Piserchio *et al.* (30), ∼252–266; Fig. S3) that lies at the interface between the two lobes of the α−kinase appears to bridge the T348 and S500 sites. Phosphorylated T348 forms several hydrogen bonds with T252 and R254, which line the PBP, on the N-terminus of αD. Most of the structures show unphosphorylated S500 engaged to the C-terminus of αD, forming hydrogen bonds with H260 and E264. The latter interaction is likely destabilized upon S500 phosphorylation (or in the S500D mutant) (30). Some structures of the CaM•eEF-2K_TR_ complex suggest disorder in the region around S500, best illustrated by those bound to an ATP-competitive inhibitor (33). These structures may indicate disorder in this region in the CaM-bound state, which is likely further enhanced in the presence of an intact R-loop, in line with the HXMS and biochemical observations. These findings support a model in which the S500-adjacent region acts as an inhibitory element that can be relieved either by Ca^2+^/CaM binding or by S500 phosphorylation. Further, dual phosphorylation at T348 and S500 may stabilize an open, CaM-bound-like conformation that lowers the energetic barrier for CaM binding and increases eEF-2K’s sensitivity to both apo- and Ca^2+^-bound CaM. Significantly, S500 phosphorylation also accelerates T348 autophosphorylation by ∼6-fold (19), reinforcing its role as a priming modification that promotes eEF-2K activation and stabilizes the active complex.

Recent studies using a CaM–eEF-2K fusion protein show that covalent attachment of the C-lobe of CaM renders kinase activity insensitive to Ca^2+^ (28), indicating that the primary role of Ca^2+^ is to enhance CaM binding. However, S500 autophosphorylation remains strictly Ca^2+^-dependent (19), suggesting that Ca^2+^ induces specific conformational changes necessary for this modification. In wild-type eEF-2K, efficient S500 autophosphorylation depends on both Ca^2+^/CaM binding and prior phosphorylation at T348. Notably, S500 autophosphorylation is significantly slower than T348 autophosphorylation (half-life ∼10 min *versus* ∼0.3 s) (19), suggesting that S500 autophosphorylation serves as a delayed regulatory step that integrates sustained Ca^2+^/CaM signaling. In contrast, PKA-mediated phosphorylation of S500 bypasses the Ca^2+^/CaM requirement, providing a mechanism by which global signals, such as nutrient status, energy stress, or cAMP pathway activation, can modulate eEF-2K function (19, 27). Under the CaM-binding conditions used here, introduction of S500D into the T348-unphosphorylated enzyme does not significantly alter Ca^2+^/CaM association, indicating that S500 phosphorylation alone does not measurably enhance CaM binding in the basal state. Instead, PKA-mediated phosphorylation of S500 is best understood as a priming modification: when S500 is phosphorylated before a Ca^2+^/CaM rise, subsequent T348 autophosphorylation is accelerated, and the dual-modified enzyme attains a stable active conformation more rapidly, thereby shortening the latency to activation and bypassing the slow, Ca^2+^/CaM-dependent S500 autophosphorylation step.

Notably, the *K*_CaM_ for apo-CaM binding to S500D-eEF-2K^*p*^ is approximately 1.5 μM (19), which lies within the physiological range of cellular CaM concentrations (2–25 μM) (34). This suggests that the doubly phosphorylated enzyme could remain responsive to CaM that is predominantly Ca^2+^-free at basal cytosolic Ca^2+^ levels (∼100 nM) (29). Beyond phosphorylation-dependent regulation, our previous work demonstrated that ADP binds to eEF-2K, enhancing its interaction with CaM (35). Together, these findings suggest that ADP may act alongside S500 phosphorylation as an allosteric modulator, promoting assembly of the active CaM•eEF-2K complex, particularly under conditions of metabolic stress or low ATP availability.

Phosphorylation at S500 has been linked to ubiquitin-mediated degradation of eEF-2K, accounting for the reduced protein levels in the S500D mutant (Fig. 4*A*) (32). This highlights a dual role for the modification: while it activates the kinase and promotes CaM binding, it may also act as a degradation signal targeting eEF-2K for proteasomal turnover. However, S500 phosphorylation alone is not sufficient to trigger degradation. Disruption of CaM binding or kinase activity restores S500D eEF-2K levels, suggesting that degradation occurs only in the context of an active eEF-2K•CaM complex (19). This mechanism may ensure precise temporal control by linking activation to self-limiting inactivation. Unlike CaMKII, whose T286 phosphorylation increases CaM affinity and promotes partial Ca^2+^-independent activity without affecting stability (36, 37, 38), S500 phosphorylation in eEF-2K appears to coordinate both activation and degradation, revealing a unique self-regulatory mechanism sensitive to cellular signals.

Previous models, informed by both autophosphorylation and PKA-mediated phosphorylation studies, recognized S500 as a modulatory site that can promote CaM-independent activity (27) and, in some cases, trigger degradation when bound to CaM in an active complex (17). Our data expand this view by revealing three additional functions: (i) enhancing CaM sensitivity and stabilizing the active state primarily in the T348-phosphorylated enzyme, (ii) disruption of an inhibitory R-loop segment near S500 to facilitate CaM binding, and (iii) showing that targeted deletions in the R-loop can mimic these effects. Together, these findings position S500 phosphorylation as a central integrator of Ca^2+^, PKA, and structural cues that coordinate eEF-2K activation, basal activity, and turnover.

**Figure 5:**
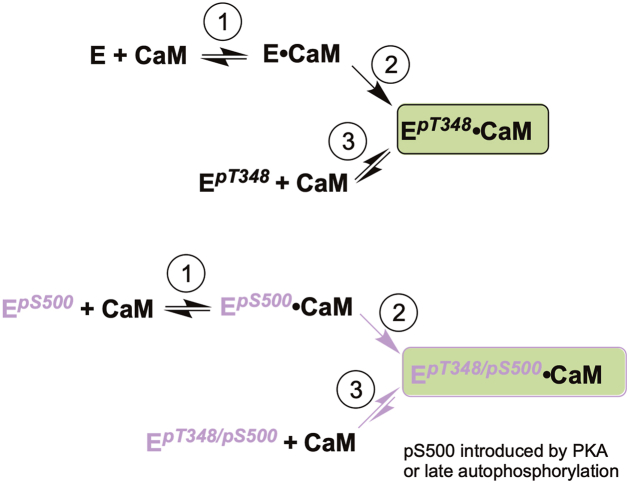
**Mechanistic model for regulation of eEF-2K by S500 phosphorylation.***Top* scheme (*black* species): Unmodified eEF-2K is denoted E (*black*). In the presence of Ca^2+^, calmodulin (CaM) binds reversibly to E (step 1) to form an E•CaM complex. Ca^2+^/CaM binding triggers rapid autophosphorylation on T348 within this bound complex (step 2), generating the active E^*pT348*^•CaM species, which exhibits increased affinity for CaM. Once T348 is phosphorylated, E^*pT348*^ can also bind CaM directly from solution (step 3), further favoring formation and lifetime of the E^*pT348*^•CaM complex. Autophosphorylation on S500 is not explicitly drawn in this scheme; it is a later, slower reaction that requires both Ca^2+^/CaM binding and prior T348 phosphorylation and therefore occurs only within the E^*pT348*^•CaM complex after steps 1 to 2. *Bottom* scheme (*purple* species): Species that are phosphorylated on S500 are depicted in purple (E^*pS500*^, E^*pT348/pS500*^). S500 phosphorylation can be introduced either by eEF-2K autophosphorylation, which occurs in the CaM-bound E^*pT34*8^•CaM complex, or by PKA, which can act independently of Ca^2+^/CaM and *p*T348. In the PKA-primed pathway, S500 is phosphorylated before a Ca^2+^/CaM signal, yielding E^*pS500*^. Ca^2+^/CaM then binds reversibly to E^pS500^ (step 1) to form E^*pS500*^•CaM, and Ca^2+^/CaM binding again drives rapid T348 autophosphorylation within this complex (step 2), allowing the enzyme to reach the dual-modified E^*pT348/pS500*^•CaM state more quickly. In the late autophosphorylation pathway, S500 is added while CaM is already bound to E^*pT348*^; CaM remains associated during S500 autophosphorylation, and subsequent CaM dissociation yields the dual-modified E^*pT348/pS500*^ species, which can then rebind CaM directly (step 3), stabilizing the E^*pT348/pS500*^•CaM state. *Purple arrows* in the *bottom* scheme highlight the steps preferentially influenced by S500 phosphorylation: acceleration of T348 autophosphorylation in the E^*pS500*^•CaM complex (step 2) and stabilization of CaM binding to the dual-modified E^*pT348/pS500*^ species (step 3). Under our binding conditions, S500 phosphorylation alone does not measurably increase CaM association in the T348-unphosphorylated enzyme; instead, *p*S500 acts mainly to accelerate T348 autophosphorylation in the CaM-bound complex and to stabilize the CaM-bound, dual-modified active state. In this model, S500 phosphorylation functions as a priming and stabilizing modification that cooperates with *p*T348 and Ca^2+^/CaM to control the timing and persistence of eEF-2K activation.

Our results support a sequential activation model for eEF-2K (Fig. 5) in which initial Ca^2+^/CaM binding promotes rapid T348 autophosphorylation, stabilizes the active conformation, and increases CaM affinity. PKA-mediated phosphorylation of S500 can occur in parallel, bypassing the Ca^2+^/CaM requirement and thereby priming the kinase. When S500 is phosphorylated (either by PKA or after T348 autophosphorylation), the enzyme shows enhanced CaM sensitivity and increased intrinsic activity, consistent with disruption of an inhibitory segment in the R-loop. We favor a model in which dual modification at T348 and S500 stabilizes an active-like state, reducing the Ca^2+^/CaM requirement for activation and increasing competitiveness for limiting CaM (and possibly apo-CaM). While we observe strong CaM-independent (“intrinsic”) activity for the recombinant, dually modified enzyme *in vitro*, whether this activity is sufficient to exert a meaningful effect on eEF-2 phosphorylation in cells is possible but uncertain and likely context-dependent (*e.g.*, local CaM abundance, ADP, and competing CaM clients). Accordingly, we propose that S500 phosphorylation primarily lowers the activation threshold for Ca^2+^/CaM, potentially permitting limited activity at basal Ca^2+^, rather than conferring robust CaM-independent signaling in cells. This dual modification also links activation to ubiquitin-mediated degradation, coupling eEF-2K’s activation to self-limiting turnover. Together, these findings redefine S500 phosphorylation as a multifunctional regulatory switch that integrates Ca^2+^ signaling, PKA inputs, and metabolic cues to fine-tune translation elongation in response to cellular needs.

While our findings provide significant mechanistic insights, further studies are needed to define the structural basis of these regulatory mechanisms fully. The absence of a full-length eEF-2K structure limits our understanding of how the intact R-loop influences the crosstalk between T348 and S500, as well as the interactions of the latter with the kinase core and potentially with CaM. Moreover, the physiological relevance of S500 phosphorylation in specific cellular contexts, such as neurons, where eEF-2K is essential for synaptic plasticity (9, 11, 39), remains unclear. Our work offers a mechanistic framework for exploring how post-translational modifications, CaM dynamics, and upstream signaling pathways converge to regulate eEF-2K function in both health and disease.

## Experimental procedures

### Purification of recombinant eEF-2K and CaM proteins

Recombinant eEF-2K, corresponding mutant proteins, and TEV protease were expressed and purified as described previously (40). Briefly, the eEF-2K vector (pET32a Trx-His_6_-eEF-2K) was grown in Rosetta-gami 2 (DE3) competent cells (Novagen) at 37 °C until the optical density reached 0.6 to 0.8. The cells were induced with 0.5 mM IPTG (Gold Bio #I2481C) and collected after 16 h of expression at 22 °C. To purify eEF-2K without phosphorylation on T348, it was co-expressed with λ-phosphatase (pCDF-Duet1). The Trx-His_6_-tagged proteins were purified from the cell lysate using Ni-NTA (Invitrogen 60-0442) affinity chromatography, and the tags were removed using 1.5% (w/w) TEV protease. The proteins were further purified using an ÄKTA pure FPLC system equipped with a Mono Q 10/100 Gl anion exchange column (Cytiva) and a HiPrep 26/60 Sephacryl S-200 HR gel filtration column (Cytiva). Purified eEF-2K was dialyzed into storage buffer (25 mM HEPES (Sigma-Aldrich #H3375), pH 7.5, 50 mM KCl (Sigma-Aldrich #P9541), 0.1 mM EDTA (Sigma-Aldrich #431788), 0.1 mM EGTA (Sigma-Aldrich #E3889), 2 mM DTT (Gold Bio #DTT100), and 10% glycerol (Fisher Bioreagents BP229-4)), concentrated, and stored at −80 °C. The phosphorylation status of T348 was confirmed by immunoblot using a phospho–T348–specific antibody (Phosphosolutions #EP4411), as described previously (19). CaM and CaM_IAE_ were purified following procedures described by Putkey and Waxham (41).

### Native gel-based CaM_IAE_ binding assay

The ability of recombinant eEF-2K proteins (0.67 μM) to bind 25 μM apo-CaM_IAE_ or 5 μM Ca^2+^/CaM_IAE_ (150 μM free Ca^2+^) was assessed by incubating samples for 20 min at room temperature in native binding buffer A [25 mM HEPES, pH 6.8, 50 mM KCl, 1 mM EGTA, 2 mM DTT, 2 mM DTT, 0.005% Brij L23 (Sigma-Aldrich #B4184), and native sample buffer (Bio-Rad #1610738)] and 0 or 1.15 mM CaCl_2_ (Honeywell #21114) in a final volume of 30 μl. The samples were loaded onto a nondenaturing gel (Bio-Rad Mini-PROTEAN TGX, 4–15%) in native running buffer (25 mM Tris (Sigma-Aldrich #T6066), 192 mM glycine (Sigma-Aldrich #G7126)) and run at 50-80V, 4 °C, protected from light. The CaM_IAE_ fluorescence was measured using a GE Amersham Imager 600 (excitation at 312 nm). The total protein was visualized by Coomassie (Thermo Scientific #20278) staining.

### CaM_A2C_ mutagenesis, expression, and purification

The N-terminal Ala of CaM (pET-23d vector) was mutated to Cys using site-directed mutagenesis (QuikChange II, Agilent). PCR was followed by DpnI digestion of the parent DNA and transformation into competent DH5α cells (ThermoFisher, #EC0112), from which the pET-23d vector containing CaM_A2C_ was purified and confirmed through Sanger sequencing. BL21 (DE3) cells (New England Biolabs, #C2527H) containing the CaM_A2C_-pET-23d vector were grown to an OD of 0.6 to 0.8 in LB media at 37 °C, and protein expression was induced with the addition of 500 μM IPTG. Expression was carried out over 5 h at 30 °C, after which the cells were collected by centrifugation.

CaM_A2C_ was purified according to the procedure reported by Long *et al.* (28), with minor modifications as described below. Cells expressing CaM_A2C_ were lysed by three consecutive freeze-thaw cycles followed by sonication in resuspension buffer [50 mM Tris (pH 7.5) with 10 mM CaCl_2_ and 0.1% BME]. The lysate was clarified by centrifugation before heat treatment, and a second centrifugation removed the heat-precipitated proteins. The resulting supernatant was filtered and loaded onto a HiPrep Phenyl FF 16/10 column (Cytiva), where the protein was washed and eluted, with 0.1% BME added to each buffer. The eluted protein was then loaded onto a HiPrep 26/60 Sephacryl S-200 HR size exclusion column (Cytiva). The protein was eluted over 1.25 column volumes (CV) of gel filtration buffer [25 mM Tris (pH 7.5), 10 mM EGTA, 150 mM NaCl, with 0.1% BME (Sigma-Aldrich #M3148)] at a flow rate of 0.8 ml/min at 4 °C. The protein was concentrated and dialyzed against 25 mM HEPES (pH 7.5) with 2 mM DTT for storage.

### Labeling CaM_A2C_ with the Cy3 fluorophore

CaM_A2C_ was extensively dialyzed against 20 mM MOPS (Sigma-Aldrich #M5162) (pH 6.8) to remove DTT. The protein was diluted to 2.5 mg/ml and incubated with a 10-fold excess of TCEP (Soltec Ventures #9879229446) (1.5 mM) for 30 min at room temperature (without mixing). A 15 mM solution of Cy3-maleimide (AAT Bioquest, #142) in DMSO was added dropwise (2 μl every 10 min) to the protein solution at room temperature, with mild agitation on an orbital shaker. Once a molar ratio of approximately 10:1 dye to protein was achieved, the solution was incubated for an additional 30 min with mild agitation before quenching with a two-fold excess of BME (2 mM). The excess fluorophore was removed by loading the sample onto a PD-10 desalting column (Cytiva) and collecting the protein in 300 μl aliquots of 20 mM MOPS (pH 6.8) with 2 mM DTT. The labeling efficiency was calculated as the ratio of the concentration determined by A_554_ to that determined by A_280_. The protein (CaM_Cy3_) was found to be approximately 15% labeled.

### Native gel detection of CaM_Cy3_ binding

Samples containing 60 nM eEF-2K (WT or S500D, with or without T348 phosphorylation, as indicated by “^*p*^” or “^λ^”, respectively) and 40 nM CaM_Cy3_ were incubated for 20 min at room temperature in native binding buffer B [20 mM MOPS, pH 6.8, 50 mM KCl, 0.1 mM EGTA, 0.15 mM CaCl_2_, 2 mM DTT, 0.005% Brij, and native sample buffer (Bio-Rad)]. 40 μl of sample was loaded onto a nondenaturing gel (Bio-Rad Mini-PROTEAN TGX, 12%) in native running buffer and run at 50-80V, 4 °C, protected from light. The CaM_Cy3_ fluorescence was measured using a Typhoon RGB imager (Cytiva) using the 532 nm laser and Cy3 emission filter. The fluorescence intensity of each lane was measured using ImageJ software, and the fluorescence of the top band was divided by the total lane fluorescence to determine the fraction of bound CaM_Cy3_. The total protein was visualized by silver staining using Silver Stain Plus (Bio-Rad, #161-0449) according to the manufacturer’s protocol.

### Kinase assays

CaM-independent intrinsic activity assays were performed by combining 400 nM eEF-2K^*p*^, 40 nM S500D-eEF-2K^*p*^, 800 nM T348A-eEF-2K, or 400 nM T348 A/S500D-eEF-2K with either a single concentration (450 μM) of the substrate peptide (PepS: Ac-RKKYKFNEDTERRRFL-Amide, Peptide 2.0), or varied concentrations of PepS in activity buffer [25 mM HEPES (pH 7.5), 2 mM DTT, 20 μg/ml BSA, 100 μM EDTA (≥99% Sigma-Aldrich #EDS), 100 μM EGTA (≥99.0% Sigma-Aldrich #0377), 10 mM MgCl_2_ (≥98% Sigma-Aldrich #M8266), 0.005% Brij L23, 50 mM KCl, and 150 μM CaCl_2_]. Reactions were initiated with 1 mM ATP (Roche #10519987001; 99% pure) spiked with [γ-^32^P]-ATP (Revvity), and time points were collected and analyzed as described previously (18). CaM-dependent assays at a single CaM concentration were carried out with 1 nM eEF-2K^*p*^ or S500D-eEF-2K^*p*^, 150 μM PepS, and 2 μM CaM in activity buffer. For single peptide concentrations, data were fit by linear regression. For PepS dependence curves, the data were fit to Equation 1.

(1) $$k_{obs}=\frac{k_{\text{cat}}^{\text{app}}\left[S\right]}{K_{m}^{\text{app}}+\left[S\right]}$$

### Hydrogen-deuterium exchange mass spectrometry measurements

CaM and eEF-2K_ΔN_ were expressed and purified using protocols described previously (42). eEF-2K_ΔN_ was treated twice with a ∼1/100 ratio of λ-phosphatase overnight at 4 °C to obtain a fully dephosphorylated species. The CaM-bound eEF-2K_ΔN_ sample was prepared by incubating eEF-2K_ΔN_ in mass spectrometry buffer containing 20 mM Tris (pH 7.5), 200 mM NaCl, 2 mM DTT, 1.0 mM CaCl_2,_ with a threefold excess of CaM in an identical buffer and then purifying the respective complexes by size exclusion chromatography, as previously described (42).

Stock solutions (∼45 μM) were diluted ∼35-fold in an otherwise identical D_2_O-based buffer (pH∗ = 7.5) and incubated for variable amounts of time at 15 °C. The exchange was quenched by diluting the sample 1/1 with a solution containing 5 M guanidinium chloride and 1% formic acid at 2 °C. The sample was then digested (7 °C, 30 s) using an Enzymate BEH Pepsin column (Waters), the resulting peptides were captured using a C8 Trap Cartridge, and then resolved using a C18 HPLC column kept at 4 °C, operated by an Ultimate 3000 Thermo-Scientific instrument in line with a maXis-II ETD ESI-QqTOF (Bruker spectrometer). Some relevant acquisition parameters are as follows: the capillary voltage was set to 4200 V, the nebulizer pressure was 1.7 Bar, the dry gas flow rate was 8.9 L/min, the drying temperature was 200 °C, and the collision energy was 4.0 eV for a 70.0 μs transfer time. The funnel 1 RF was 250 Vpp, the multipole RF was 300 Vpp, and the quadrupole ion energy was 2.0 eV. MS spectra were collected at 1.2 Hz and MS/MS spectra at 5 Hz, with an acquisition cycle of 3 s and a mass range of 100 to 2250 m/z. Additional experimental details have been published elsewhere (42). Reference spectra were obtained by diluting the samples in a H_2_O-based buffer and then collecting MS/MS data, which were subsequently analyzed using Bruker COMPASS and BIOTOOLS software packages. HDExaminer 3.4.2 (Sierra Analytics) was used to assess ^2^H-incorporation. Two datasets were collected on different dates using similar but distinct samples. In the first data collection, a single experiment was recorded for the following time points: eEF-2K_ΔN_: 30, 300, 1000, and 5000 s; CaM•eEF-2K_ΔN_ complex: 30, 300, 1000, and 3000 s. In the second data collection, two time points (30 s, 300 s) were collected in triplicate for all samples. The overall peptide coverage for CaM and eEF-2K_ΔN_ was 88.5% and 64.5%, respectively.

### Mutant eEF-2K activity in MCF10A cells

Wild-type and mutant eEF-2K (cloned into plasmid 10,792; Addgene) were transfected into MCF10 A *eef2k*^−/−^ cells (Sigma-Aldrich, #CLLS1051) as previously described (17). 24 h after transfection, cells were lysed, and protein concentration was determined using a Pierce BCA assay (Thermo Fisher Scientific, #T23227). 30 μg of protein was then analyzed by SDS-PAGE and western blotting as described previously (17). For western blotting, the following antibodies were used: eEF-2K (C-12) (Santa Cruz Biosciences, #sc-390710); eEF-2 (Cell Signaling, #2332); phospho-eEF-2 (Cell Signaling, #2331); Pan-actin (Cell Signaling, #8456); IRDye 800CW Goat anti-Rabbit (LiCor, #926-32211); IRDye 680RD Goat anti-Mouse (LiCor, #926-68070); and Goat Anti-Rabbit IgG (H+L)-HRP Conjugate (Bio-Rad, #1721019). Total eEF-2K, eEF-2, and actin were visualized *via* the LI-COR Odyssey Sa instrument. Phospho-eEF-2 was visualized using WesternBright ELC (Advansta, #K-12045-D20) and Amersham Imager 600 (GE Healthcare Life Sciences). All cell cultures used in this study were confirmed to be mycoplasma-free by routine testing.

### Data availability

The HXMS data were deposited in the MassIVE repository with accession code MSV000099717. All other supporting evidence for the findings of this study is available within the article and in the supporting information.

## Supporting information

This article contains supporting information.

## Conflict of interest

The authors declare that they have no conflicts of interest with the contents of this article.
