# Supplementary material for: Ser500 phosphorylation acts as a conformational switch to prime eEF-2K for activation
Source: J Biol Chem. 2025 Dec 22;302(2):111087. doi: 10.1016/j.jbc.2025.111087 (PMC12834908; doi:10.1016/j.jbc.2025.111087)
Supplement: Supporting information [file mmc1.docx]

**SUPPORTING INFORMATION**

**for**

**Ser500 phosphorylation acts as a conformational switch to prime eEF-2K for activation**


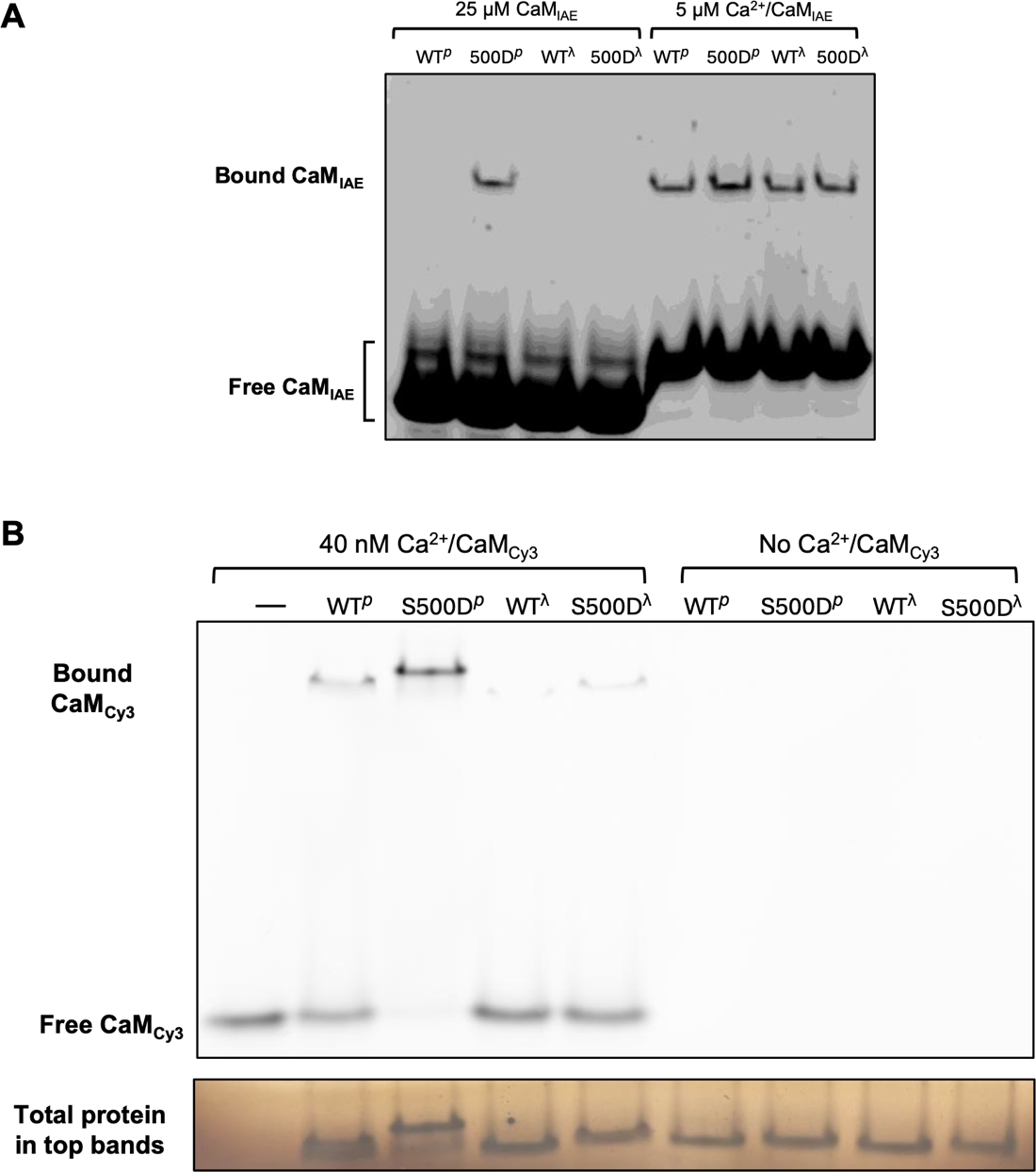


**Fig. S1 Native gel binding assays with fluorescent CaM. (A)** The entire gel from Fig. 1A, showing bound and unbound CaM_IAE_ fluorescence (312 nm excitation). The bound CaM_IAE_ co-migrates with recombinant eEF-2K (~82 kDa) on the native gel, while the excess free CaM (~17 kDa) migrates more rapidly through the gel. The addition of Ca^2+^ causes a slight upward shift in free CaM. **(B)** Top panel: Image of the entire gel from Fig. 1B visualized using the Cy3 channel on an Amersham Typhoon RGB imager. The left five lanes include the indicated eEF-2K construct and Ca^2+^/CaM. The right four lanes contain only the indicated eEF-2K construct. Bottom: Silver stain of eEF-2K to demonstrate consistent loading via Amersham™ Imager 600 (GE Healthcare Life Sciences).

##### **
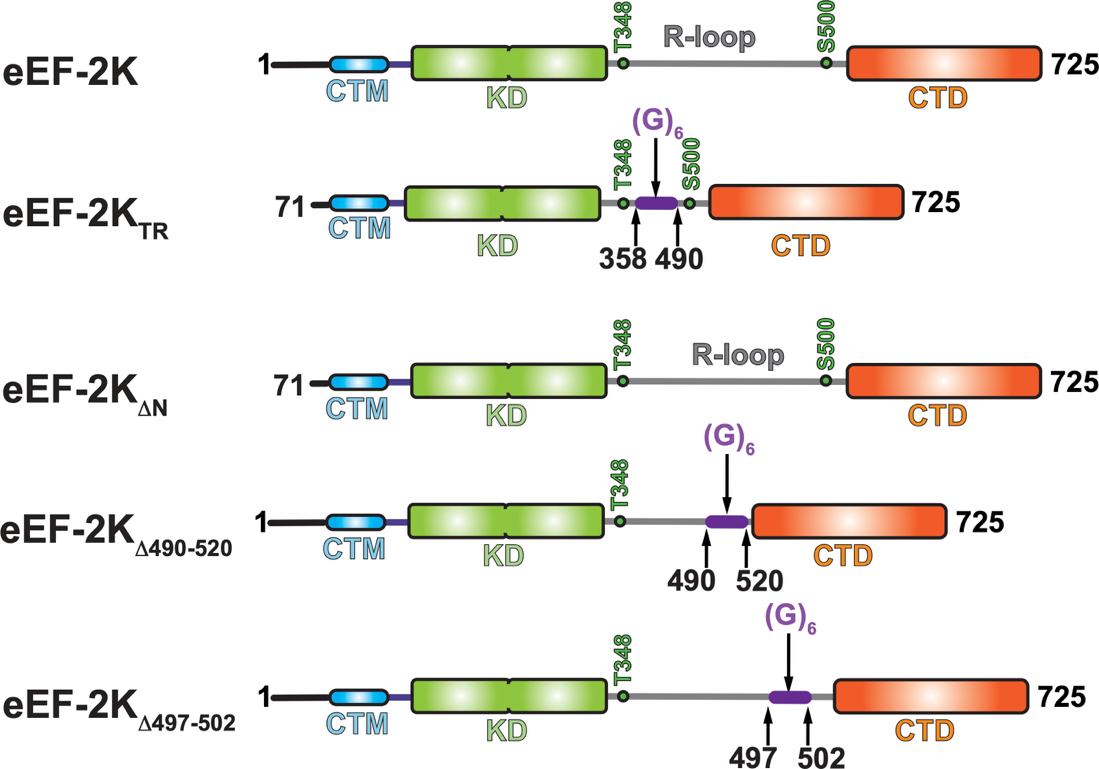
**

**Fig. S2** **eEF-2K constructs used in this study.** Schematic representation of the various eEF-2K constructs, including wild-type full-length eEF-2K, eEF-2K_TR_ [70 N-terminal residues are absent, and a 6-glycine linker has replaced the 359-490 segment of the regulatory loop (R-loop); the linker is indicated in purple], eEF-2K_ΔN_ (missing 70 N-terminal residues but containing an intact R-loop), eEF-2K_Δ490-520_ (a 6-glycine linker has replaced 490-520 segment of the R-loop), and eEF-2K_Δ497-502_ (a 6-glycine linker has replaced 497-502 segment of the R-loop). The N-terminal calmodulin-targeting motif (CTM), the α−kinase domain (KD), the R-loop, and the C-terminal domain (CTD) are indicated. The activating T348 and S500 sites are located at the two ends of the R-loop.


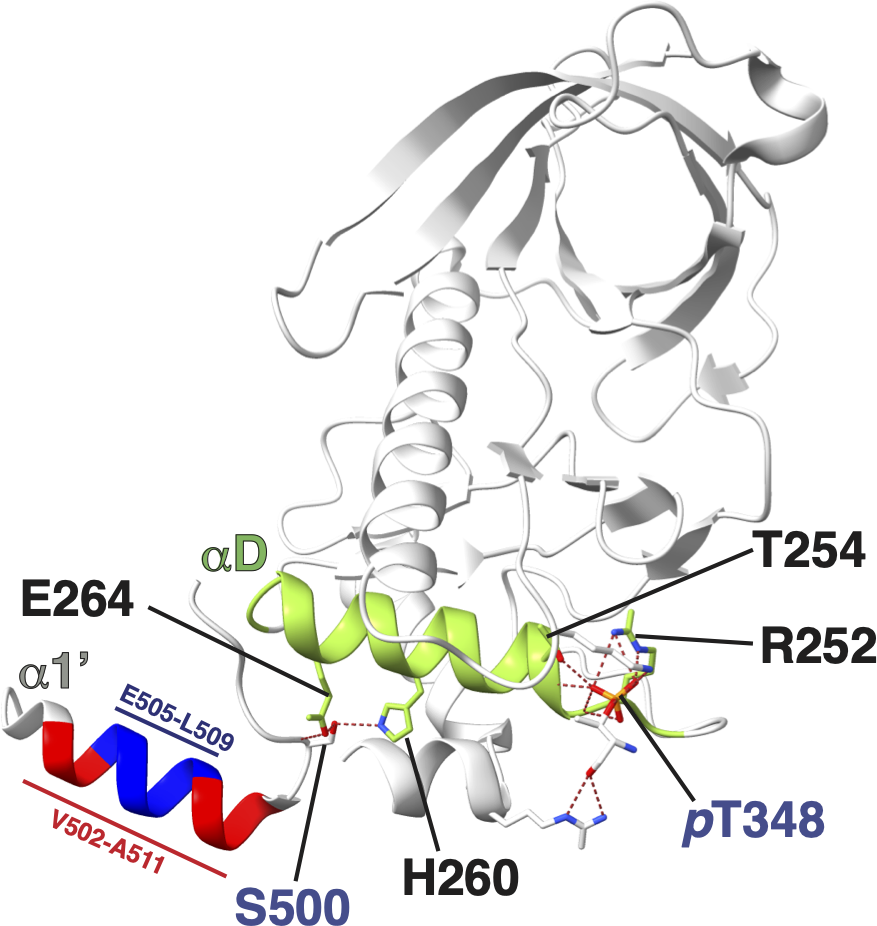


**Fig. S3 A helix at the interface between the N- and C-lobes of the kinase domain of eEF-2K likely links the T348 and S500 phosphorylation sites.** The αD helix (252-266, green; the rest of the KD, 110-320 in grey) appears to bridge the T348 and S500 sites. Phosphorylated T348 (*p*T348) docks into the phosphate-binding pocket, forming hydrogen bonds with two residues, R252 and T254, that lie on the N-terminus of αD. In most structures of the CaM•eEF-2K_TR_ complex obtained thus far, unphosphorylated S500, that lies at the N-terminal end of a helical segment (α1’), docks into a shallow pocket, forming hydrogen bonds with H260 and E264 on the C-terminus of αD. The two peptides that show the most substantial statistically significant changes in the HXMS studies (Fig. 3) are derived from α1’; E505-L509 is colored blue, and V502-A511 is colored red). While this configuration of α1’ and its proximal regions is seen in most structures, there is evidence of considerable disorder in this region (as indicated by elevated B-factors). In one of the structures of the CaM•eEF-2K_TR_ complex bound to the ATP-competitive A-484594, this region, including S500, could not be modeled. The presence of an intact R-loop (as in eEF-2K_ΔN_) would likely further destabilize this region in the CaM-bound state, as suggested by the HXMS analysis. It is worth noting that phosphorylation of S500 would likely destabilize its interaction with E264, seen in the structure of the CaM•eEF-2K_TR_ complex, if such an interaction indeed exists in solution. We expect that a crystal structure of the S500D mutant, when available, will resolve this issue.
